# Classical (Local and Contextual) Probability Model for Bohm–Bell Type Experiments: No-Signaling as Independence of Random Variables

**DOI:** 10.3390/e21020157

**Published:** 2019-02-08

**Authors:** Andrei Khrennikov, Alexander Alodjants

**Affiliations:** 1 Mechanics and Optics (ITMO) Department, National Research University for Information Technology, St. Petersburg 197101, Russia; 2 International Center for Mathematical Modeling in Physics and Cognitive Sciences, Linnaeus University, SE-351 95 Växjö, Sweden

**Keywords:** quantum versus classical probability, Bohm–Bell type experiments in physics and psychology, localty, contextual hidden-variables models, (no-)signaling, random generators, selection of experimental settings, conditional probability

## Abstract

We start with a review on classical probability representations of quantum states and observables. We show that the correlations of the observables involved in the Bohm–Bell type experiments can be expressed as correlations of classical random variables. The main part of the paper is devoted to the conditional probability model with conditioning on the selection of the pairs of experimental settings. From the viewpoint of quantum foundations, this is a *local contextual hidden-variables model.* Following the recent works of Dzhafarov and collaborators, we apply our conditional probability approach to characterize (no-)signaling. Consideration of the Bohm–Bell experimental scheme in the presence of signaling is important for applications outside quantum mechanics, e.g., in psychology and social science. The main message of this paper (rooted to Ballentine) is that quantum probabilities and more generally probabilities related to the Bohm–Bell type experiments (not only in physics, but also in psychology, sociology, game theory, economics, and finances) can be classically represented as conditional probabilities.

## 1. Introduction

This paper is directed to resolution of the old foundational problem of quantum mechanics: *whether it is possible to represent quantum states by classical probability (CP) distributions and quantum observables by random variables* [1]. In fact, we analyze the general measurement scheme involving compatible and incompatible observables which need not be described by the quantum formalism. However, our starting point is construction of the CP-representation for quantum mechanics.

Throughout the paper, we use capital Latin letters, A,B,R (with indexes) to denote observables and small letters a,b,r (with indexes) to denote classical random variables (RVs).

### 1.1. Towards CP-Representation

The first CP-representation of quantum mechanics based on of *symplectic tomogram* was constructed in works [2,3,4]. Another construction of the CP-representation of quantum mechanics is based on so-called *prequantum classical statistical field theory* [5,6,7,8,9,10]. It should be honestly said that the tomographic and random field approaches were practically ignored by the quantum foundational community. Since the first days of quantum mechanics, it was commonly believed that CP-theory, see Kolmogorov [11], cannot serve to represent incompatible quantum observables. At the early stage of development of quantum theory, this belief was firmly based on *the Heisenberg uncertainty principle.* By the straightforward interpretation of the Heisenberg uncertainty relation, position and momentum cannot be jointly assigned to an individual quantum system. Under such an interpretation, it was meaningless even to speak about the joint probability distribution (jpd) for position and momentum. For example, for Wigner [12], p. 749, it was clear that

“In quantum theory there does not exist any similar simple expression for the probability because one cannot ask for the simultaneous probability for the coordinates and momenta.”

Here “similar” is related to the case of classical statistical mechanics and the Gibbs–Boltzmann formula for statistical equilibrium. We also cite Feynman [13] (italic shrift was added by the authors of this paper):

“From about the beginning of the twentieth century experimental physics amassed an impressive array of strange phenomena which demonstrated the inadequacy of classical physics. The attempts to discover a theoretical structure for the new phenomena led at first to a confusion in which it appeared that light and electrons sometimes behaved like waves and sometimes like particles. This apparent inconsistency was completely resolved in 1926 and 1927 in the theory called quantum mechanics. The new theory asserts that there are experiments for which the exact outcome is fundamentally unpredictable, and that, in these cases, one has to be satisfied with computing probabilities of various outcomes. *But far more fundamental was the discovery that in nature the laws of combining probabilities were not those of the classical probability theory of Laplace.*”

### 1.2. No-Go Statements

The main argument against the possibility to proceed with the CP-representation is based on *no-go theorems.* The first no-go theorem was proven by von Neumann [14] (German edition -1932): the theorem on nonexistence of dispersion free states. This theorem was strongly criticized by Bell [15] who pointed to non-physicality of von Neumann’s rule for correspondence between classical and quantum probabilistic structures:

- probabilities→states,
- random variables→ Hermitian operators,

cf. Section 2.3, Section 3.4. Bell’s own no-go theorem [15,16] has much better reputation than von Neumann’s theorem. It has a very big impact on quantum foundations, quantum information, and quantum technology. At the same time, it generated a plenty of critical papers (see, e.g., [17,18,19,20,21,22,23] for some resent publications). Bell proposed the CP-description of the Bohm–Bell type experiments. This approach is known as the hidden-variables description. Since it is very difficult to test experimentally the original Bell inequality (see [24,25] for a discussion), Clauser, Horne, Shimony, and Holt (CHSH) [26] modified Bell’s approach on the basis of the CHSH-inequality. We denote the CP-model proposed by them by the symbol $M_{BCHSH}$ (see Section 2.2). We remark that, in spite of a rather common opinion, this modification is not equivalent to the original Bell approach (see [27] for a discussion and comparison of the original Bell and CHSH-inequality).

Fine [28,29] showed that the CHSH-inequality is satisfied if and only if the assumption on the existence of the jpd (for the four observables $A_{1},A_{2},B_{1},B_{2}$ involved in the experiment—see Section 2.1) holds true. The latter is equivalent to using CP-theory. Therefore, one can conclude that a violation of the CHSH-inequality inequality by quantum probabilities implies inapplicability of the CP-theory for description of quantum observables.

Nevertheless, as was shown by Khrennikov and coauthors [30,31] and by Dzhafarov and coauthors [32,33,34,35,36,37,38], the Bohm–Bell type experiments can be modeled with the aid of the CP-representation of quantum observables. However, such CP-models are not so straightforward as $M_{BCHSH}.$ In this paper, we present a general CP-model based on the conditional probability scheme which was explored in [31] (in the very concrete situation). Denote the models developed in [32,33,34,35,36,37,38] and in the present article by the symbols $M_{DZ}$ and $M_{KH},$ respectively.

After the first version of this paper was submitted to arXiv [39], Khrennikov received an email from Czachor providing information about his paper [40]. This paper contains a CP-based example of violation of the CHSH-inequality. Unfortunately, Czachor’s paper did not attract so much attention and it was practically forgotten. We also point to the recent paper of by Dzhafarov and Kon [38] on general applicability of CP in quantum physics and on Khrennikov’s comment on this paper [41].

We now turn to Fine’s theorem. Fine showed that the following two statements are equivalent:

1. There is one jpd for all observables of the experiment.
2. There is a deterministic hidden-variables model for the experiment.

We postpone the general discussion on the relation of our CP-model $M_{KH}$ to the hidden-variables theory to Section 5. Here, we just stress that Fine’s statement is about noncontextual hidden variables models. Our model is deterministic, but contextual. Thus, Fine’s theorem is inapplicable to it.

### 1.3. Can Experimental Violation of Bell Type Inequalities Be Checked in the Absence of Classical Probabilistic Representation?

We point out that *CP-models for quantum experiments are important for justification of applicability of the methods of classical statistics to analyze experimental data collected in quantum physics.* We stress that, to check statistical significance of a violation of a Bell type inequality, experimenters always use classical mathematical statistics, e.g., *p*-values or Chebyshov inequality, e.g., [42,43,44,45,46,47]. However, by demonstrating that a violation of this Bell type inequality is statistically significant, one has to remember that the standard CP-representation based on model $M_{BCHSH}$ is impossible by Fine’s theorem. Therefore, the preceding CP-based statistical analysis justifying the hypothesis on the experimental violation of the Bell type inequality was meaningless. We understood that this is the strong claim. We are not able to proceed further with its justification. This problem deserves a separate study.

Of course, one can appeal to the quantum theory of decision-making. However, such appealing is meaningless for comparing classical and quantum descriptions. In contrast, with the CP-model $M_{KH}$ (or other CP-models presented, e.g., in [1,2,3,4,32,33,34,35,36,37,38]), one can apply the standard methods of (CP-based) statistics. Although these models are different both from the foundational and technical viewpoints, they can serve the same purpose. In particular, analysis of data with the aid of model $M_{KH}$ can be used to justify statistical significance of violation of the CHSH-inequality for experimental probabilities which are interpreted as classical probabilities conditional on selection of experimental settings.

### 1.4. Conditional Probability Approach

The basic distinguishing feature of $M_{KH}$ takes into account the *conditional nature* of quantum probabilities. Generally, we follow Ballentine [48,49], especially his paper [50]. In the present paper, conditioning is modeled with the aid of the *random generators selecting the experimental settings.* They are represented as random variables (RVs) $r_{a},r_{b}$ which are supplementary to the “basic” RVs $a_{1},a_{2},b_{1},b_{2}$ (see Section 2.2, Section 3.2). These RVs are absent in $M_{BCHSH}.$ At the same time, the random generators play the crucial role in the real experimental design of such experiments. (These are physical devices generating random numbers or computers generating pseudo-random numbers.) Hence, their mathematical realization by RVs has to added to $M_{BCHSH}.$ We remark that Bohr emphasized that, in modeling quantum phenomena, all components of the experimental arrangement should be taken into account [51,52,53,54,55]. Thus, ignoring RVs representing mathematically the random generators makes a model without them (as, e.g., $M_{BCHSH})$ inadequate for the real physical situation.

We remark that, in Khrennikov’s work [31], the random generators were also present and played the fundamentals, but not explicitly as in the present paper, in model $M_{KH}.$ The explicit presentation of random generators in $M_{KH}$ makes the structure of probability space more complicated. However, this increase of mathematical complexity is compensated by clarification of physics behind $M_{KH}.$

Other models mentioned in this paper (see [1,2,5,6,7,8,15,16,17,18,19,20,23,24,25,26,28,29,32,33,34,35,36,37,38,40] are based on unconditional probability representation. (Although prequantum classical statistical field theory is based on unconditional probabilities, to connect it with the quantum mechanical observations, conditioning on coincidence detection has to be used [9,10]). Ballentine [48,49] emphasized the role of conditional probabilities in quantum mechanics, but without presenting a formal mathematical model. We remark that, in the first part of his analysis of the two slit experiment, Feynman [13] discussed its contextual structure. However, he did not proceed to a formal contextual probabilistic model. He used the path integral formalism to explain a violation of additivity of probability.

Finally, we point to a series of papers on nonclassical conditioning (“contextual probability theory”) and its applications to quantum physics and psychology [56,57,58,59,60,61,62,63,64]. This approach was based on operating with a bunch of Kolmogorov probability spaces related to experimental contexts and coupled via (generally nonclassical) conditioning. In the contextual probability framework, it is evident that the Bell type inequalities can be violated. The main point of this paper (see also [31]) is that such inequalities can be violated even with CP-conditioning, and such CP-models can be local and realistic.

### 1.5. CP-Representations in the Presence of Signaling

Model $M_{DZ}$ is based on contextual coupling of RVs corresponding to the choice of experimental settings. This model was applied to study *contextuality* in the CP-framework with the especial emphasis of the possibility to proceed in the presence of *signaling* [32,33,34,35,36,37,38]. (Contextuality studied by Dzhafarov and the coauthors is the natural extension of the notion of quantum contextuality based on the Bell type tests.) We remark that signaling is absent in quantum mechanics. Therefore, contextuality theory, developed in [32,33,34,35,36,37,38] and known as *contextuality by default* (CbD), is more general than the standard theory of quantum contextuality. In particular, the standard Bell type inequalities are modified by including the signaling contribution. They are known as the *Bell–Dzhafarov–Kujala* (BDK) inequalities. This generality provides the possibility to apply CbD outside physics, especially in psychology [63,64,65,66], where the condition of no-signaling is generally violated [33,36]. From the quantum foundation viewpoint, CbD is about a special class of (generally) nonlocal contextual hidden-variables models (see Section 5).

Refs. [30,31] aimed to show the existence of the CP-representation for the Bohm–Bell experiment with genuine quantum systems. In these papers, model $M_{KH}$ was presented in the very concrete framework coupled to classical versus quantum discussion on the CHSH-inequality. This rigid coupling with quantum mechanics led to ignoring the possibility to use model $M_{KH}$ even in the presence of signaling. Consistent CP-treatment of (no-)signaling in model $M_{DZ}$ motivated the authors of this paper to analyze (no-)signaling issue on the basis of $M_{KH}.$ In addition, we found very clear CP-interpretation of no-signaling: *independence of RVs $a_{1},a_{2},r_{a}$ representing Alice’s observables and random generator from RV $r_{b}$ representing the random generator for selecting Bob’s observables.* Thus, no-signaling has clear probabilistic meaning. In contrast to Refs. [30,31], in this paper, we proceed in a very general abstract framework which can be used both in physics and outside it, e.g., in psychology. (see [63,64,65,66] for consideration of the Bell type inequalities in psychology.)

## 2. Bohm–Bell Type Experiment: Traditional Description

In this paper, we restrict our consideration to deterministic models with hidden variables (Section 5). We recall that, in a deterministic hidden variables theory, the value of the hidden variable uniquely determines the measurement result. Stochastic models with hidden variables were invented to proceed as generally as possible. Furthermore, such generality is important for “no-go statements”. In this paper, we are concentrated on “yes-statements.”

### 2.1. Description of (Four) Observables

In the observational framework for the Bohm–Bell type experiments, *four observables* $A_{1},A_{2},B_{1},B_{2}$ are considered taking values ±1. It is assumed that the pairs of observables $(A_{i},B_{j}),i,j=1,2,$ can be measured jointly, i.e., *A*-observables are compatible with *B*-observables. However, the observables in pairs $A_{1},A_{2}$ and $B_{1},B_{2}$ are incompatible, i.e., they cannot be jointly measured. Thus, probability distributions $p_{A_{i}B_{j}}$ are well defined theoretically by quantum mechanics and they can be verified experimentally; probability distributions $p_{A_{1}A_{2}}$ and $p_{B_{1}B_{2}}$ are not defined by quantum mechanics and, hence, the question of their experimental verification does not arise.

We stress that, although our starting point is quantum mechanics and the Bohm–Bell experiment for measurement of spin of electrons or polarization of photons, we need not restrict our scheme to quantum observables. It is applicable to any measurement design involving compatible and incompatible observables—see, e.g., [63,64,65,66] for such experimental design in psychology. Here, compatibility (incompatibility) is understood as the possibility (impossibility) of joint measurement and determination of jpd.

### 2.2. Classical Probability Model (BCHSH) for the Bohm–Bell Experiment: Four Random Variables

Let (Λ,F,P) be some probability space [11]. Here, Λ is the set of hidden variables (or in mathematics“elementary events”), F is a σ-algebra of events, and *P* is a probability measure on F. We remark that, if Λ is finite, then F is the collection of all its subsets. In CP-modeling, with the CHSH framework, it can be assumed that Λ is finite. Consider two pairs of random variables $a_{1},a_{2}:\Lambda \to \left\{\pm 1\right\}$ and $b_{1},b_{2}:\Lambda \to \left\{\pm 1\right\}.$ These random variables are associated with observables $A_{1},A_{2},B_{1},B_{2}.$ This is the Bell type CP-model for the observational framework presented in Section 2.1. Denote this CP-model by $M_{BCHSH}$ (see [26]). This is a deterministic model with hidden variables and hence it is realistic. This model is also local. Following Bell [67] and Einstein [68], Clauser, Horne, Shimony, and Holt defined locality [26], p. 881, as the possibility to represent observables $A_{1},A_{2}$ by one-indexed RVs $a_{1},a_{2}.$ In a nonlocal model, a measurement of observable $A_{i}$ jointly with a measurement of observable $B_{j}$ should be represented by a double indexed RV $a_{ij}$ (see Section 5 for further details).

We remark that the jpd of four random variables $a_{1},a_{2},b_{1},b_{2}$ is well defined:

$$P_{a_{1}a_{2}b_{1}b_{2}}\left(\alpha_{1},\alpha_{2},\beta_{1},\beta_{2}\right)$$

$$=P(\lambda :a_{1}\left(\lambda \right)=\alpha_{1},a_{2}\left(\lambda \right)=\alpha_{2},b_{1}\left(\lambda \right)=\beta_{1},b_{2}\left(\lambda \right)=\beta_{2}),$$

where $\alpha_{i},\beta_{j}=\pm 1.$

In model $M_{BCHSH},$ one can form the CHSH linear combination of the correlations of the pairs of random variables $a_{i},b_{j}$

(1) $$B=\langle a_{1}b_{1}\rangle -\langle a_{1}b_{2}\rangle +\langle a_{2}b_{1}\rangle +\langle a_{2}b_{2}\rangle$$

and prove the CHSH-inequality:

(2) |B|≤2.

Here,

(3) $$\langle a_{i}b_{j}\rangle \equiv E\left(a_{i}b_{j}\right)=\int_{\Lambda }a_{i}\left(\lambda \right)b_{j}\left(\lambda \right)dP\left(\lambda \right)=\sum_{\alpha ,\beta }\alpha \beta P_{a_{i}b_{j}}\left(\alpha ,\beta \right).$$

We remark that probabilities for the joint measurements of *a* and *b* observables can be represented as the marginal probabilities for the quadruple jpd, e.g., $P_{a_{1}b_{1}}\left(\alpha ,\beta \right)=\sum_{x,y}P_{a_{1}a_{2}b_{1}b_{2}}\left(\alpha ,x,\beta ,y\right).$ This representation plays the crucial role in the derivation of CHSH-inequality (2). Moreover, by Fine’s theorem [28,29], the existence of the jpd is equivalent to satisfying the CHSH-inequality. In principle, we can select Λ as the set of vectors $\lambda =(\alpha_{1},\alpha_{2},\beta_{1},\beta_{2})$ with coordinates ±1. Here, probability *P* is given by jpd; events are all possible subsets of this Λ.

Now consider the observational probabilities $p_{A_{i}B_{j}}.$ The BCHSH-coupling between the observational and CP descriptions is straightforward; they will be presented in the next section.

### 2.3. BCHSH-Rule for Correspondence between Observational and Classical Probabilities

The observational framework (Section 2.1) is coupled with CP-model $M_{BCHSH}$ by the following correspondence rule:

*The observational probabilities $p_{A_{i}B_{j}}$ are identified with the CP-probabilities $P_{a_{i}b_{j}}.$*

This coupling leads to contradiction because the CHSH linear combination composed of observational correlations (either experimental or quantum theoretical):

(4) $$B_{\mathrm{observational}}=\langle A_{1}B_{1}\rangle -\langle A_{1}B_{2}\rangle +\langle A_{2}B_{1}\rangle +\langle A_{2}B_{2}\rangle$$

can violate CHSH-inequality (2); generally,

(5) $$|B_{\mathrm{observational}}|>2.$$

One can conclude that *CP-model $M_{BCHSH}$ is not adequate either to the quantum theoretical model or to the experimental situation.* This mismatching related to concrete CP-model $M_{BCHSH}$ and the BCHSH correspondence rule is commonly interpreted too generally:

*As the impossibility of the CP-description of quantum phenomena, the impossibility to represent quantum states by probability measures and quantum observables (generally incompatible) by classical random variables.*

### 2.4. Missed Component of Experimental Arrangement

In the CHSH observational framework, the correlations composing quantity $B_{\mathrm{observational}}$ cannot be measured jointly. The concrete experiment can be performed only for one fixed pair of indexes (i,j), experimental settings (orientations of PBSs). Generally, these settings are selected randomly by using two random generators $R_{A}$ and $R_{B}$ taking values 1,2. What are the theoretical counterparts of these random generators in $M_{BCHSH}?$ They are absent. Thus, CP-model $M_{BCHSH}$ is inadequate for the observational framework. One sort of randomness, namely generated by $R_{A},R_{B}$, is missed. We shall present another CP-model corresponding to the real experimental situation: the observational BCHSH-framework (Section 2.1) with supplementary observables $R_{A},R_{B}.$ By proceeding in this way, we follow the Copenhagen interpretation of quantum mechanics. Bohr always emphasized: *all components of the experimental arrangement (context) have to be taken into account* [51,52,53,54,55]. In addition, random generators are the important components of the experimental design, for the Bohm–Bell type tests. However, these generators are absent in the standard observational framework for the Bohm–Bell type experiments and in hidden variables model $M_{BCHSH}$ (see Section 2.1, Section 2.2). In the real physical experiments, settings of PBSs are selected with the aid of random generators. This selection process is absent in the CHSH-model. The CHSH-model is not a mathematical model of the real random experiment, but a model of four different experiments.

There are a plenty of publications on the role of random generators in confronting local realism and quantum mechanics (see, e.g., [45,69,70,71,72,73,74,75]). In terms of the foundational and experimental studies on the impact of the random generators in the Bohm–Bell type experiments, the above discussion is about

**A randomness condition:** *The inputs that we give to Alice and Bob to select experimental settings must be random. By this, we mean that Alice and Bob cannot predict the inputs that they will receive and thus adapt their strategy to the future values of the inputs.*

This randomness condition is also called the *measurement-independence or freedom of choice loophole.* The most consistent presentation of this issue can be found in the short paper of Pironio [74]. In particular, following Bell, he explains why, without equipping the Bohm–Bell experiment by a random generation of settings, a violation of the CHSH inequality has no impact.

## 3. Bohm–Bell Type Experiments: Taking into Account Random Generators

At the observational level, we plan to complete the standard description of the Bohm–Bell type experiments (Section 2.1) by taking into account the aforementioned extra components of the experimental arrangement”. Then, we shall construct a CP-model which will be adequate for the completed observational framework. It will take into account “extra components of randomness”. Denote such a CP-model under construction by $M_{\mathrm{KH}}.$

### 3.1. Description of (Six) Observables

Following Bohr, we treat random generators $R_{A}$ and $R_{B}$ as a part of experimental arrangement. Instead of the observational framework with four observables (Section 2.1) $A_{1},A_{2},B_{1},B_{2},$ we consider the framework with six observables $A_{1},A_{2},B_{1},B_{2},R_{A},R_{B}.$ The latter two observables are compatible, i.e., they can be jointly measurable; moreover, they are compatible with each of four “basic observables” $A_{1},A_{2},B_{1},B_{2}$ (see [76] for the mathematical representation of these six observables within the quantum operator formalism). In principle, in the real experimental situation, one can assume that observables $R_{A}$ and $R_{B}$ are independent. For the moment, we proceed without this assumption.

To improve the visibility of the role of random generators, in physics, we can consider the experimental design of the pioneer experiment performed by Aspect (see [77]). In the modern experimental design, there are two beam splitters, one on the *A*-side and another on the *B*-side, and two devices for random selection of orientations on the corresponding sides. Aspect considered four beam splitters and two switchers preceding corresponding pairs of beam splitters. The *A*-switcher selects randomly one of the beam splitters on the *A*-side; the *B*-switcher selects randomly one of the beam splitters on the *B*-side (switchers open optical channels to corresponding beam splitters). For this design, it is natural to introduce the additional value of observables, we set $A_{i}=0$ ($B_{j}=0)$ if its input channel is closed by the random switcher.

We consider the ideal experiment with 100 % of efficiency of the whole experimental scheme, i.e., including detector, beam splitters, an optical fibers.

Finally, we remark that typically it is claimed that $R_{A}$ and $R_{B}$ should be *quantum random generators* (see [45,69,70,71]). Thus, $R_{A}$ and $R_{B}$ should be treated as quantum observables. Therefore, it would be strange if these quantum observables were not counted as a part of the experimental arrangement (see Bohr [51]).

### 3.2. Complete CP-Model: Six Random Variables

Let again (Λ,F,P) be some probability space. We want to introduce random variables $a_{1},a_{2},b_{1},b_{2}$ associated with observables $A_{1},A_{2},B_{1},B_{2},$ but not so straightforwardly as in $M_{\mathrm{BCHSH}}.$ Additionally, we consider two random variables $r_{A},r_{B}:\Lambda \to \left\{1,2\right\}$ associated with the random generators. Besides values ±1, random variables $a_{1},a_{2},b_{1},b_{2}$ can take the value zero. The zero-value is determined by governing selections of measurement settings, i.e., $A_{1},A_{2},B_{1},B_{2},$ by random generators $R_{A}$ and $R_{B}.$ In our CP-model, it has the form:

- $a_{i}=0$ (with probability one), if the *i*-setting was not selected, i.e., $r_{A}\neq i;$
- $b_{j}=0$ (with probability one), if the *j*-setting was not selected, i.e., $r_{B}\neq j.$

We remark that in our model the zero-value has nothing to do with detection’s inefficiency (as is often considered in modeling the Bohm–Bell experiment). We model the experimental situation with detectors having 100% efficiency.

### 3.3. Constraints on Joint Probabilities Implied by Matching Condition

In terms of probability, the condition of $a-r_{a}$ matching can be written as follows:

(6) $$P(a_{i}=0|r_{a}=j)=1,i\neq j.$$

It implies that

(7) $$P(a_{i}=\alpha |r_{a}=j)=0,\alpha =\pm 1,\;i\neq j.$$

Thus, RV $a_{i}$ cannot take values ±1 if $r_{a}\neq i.$ This is the CP-presentation of the impossibility to measure observable $A_{i}$ if random generator $R_{A}\neq i.$ Equality (7) implies

(8) $$P(a_{i}=\alpha ,r_{a}=j)=0,\alpha =\pm 1,i\neq j.$$

In the same way, the condition of $b-r_{a}$ matching can be written as follows:

(9) $$P(b_{i}=0|r_{b}=j)=1,i\neq j.$$

This condition implies

(10) $$P(b_{i}=\beta ,r_{b}=j)=0,\beta =\pm 1,i\neq j.$$

From equalities (6), (9), we obtain

(11) $$P\left(a_{i}=0,r_{a}=j\right)=P\left(r_{a}=j\right),\;P\left(b_{i}=0,r_{b}=j\right)=P\left(r_{b}=j\right),i\neq j.$$

In turn, these equalities imply

(12) $$P\left(a_{i}=0,r_{a}=i\right)=P\left(r_{a}=i\right),\;P\left(b_{i}=0,r_{b}=i\right)=P\left(r_{b}=i\right).$$

The jpd of six random variables $a_{1},a_{2},b_{1},b_{2},r_{A},r_{B}$ is well defined:

$$P_{a_{1}a_{2}b_{1}b_{2}r_{a}r_{b}}\left(\alpha_{1},\alpha_{2},\beta_{1},\beta_{2},\gamma_{1},\gamma_{2}\right)$$

$$=P(\lambda :a_{1}\left(\lambda \right)=\alpha_{1},a_{2}\left(\lambda \right)=\alpha_{2},b_{1}\left(\lambda \right)=\beta_{1},b_{2}\left(\lambda \right)=\beta_{2},r_{A}\left(\lambda \right)=\gamma_{1},r_{B}\left(\lambda \right)=\gamma_{2}),$$

where $\alpha_{i},\beta_{j}=0,\pm 1,\gamma_{k}=1,2.$ The matching condition implies that, e.g., $P_{a_{1}a_{2}b_{1}b_{2}r_{a}r_{b}}(\alpha_{1},\pm 1,$ $\beta_{1},\beta_{2},1,\gamma_{2})=0.$ Thus, only 16 components of the jpd are different from zero:

$$P_{a_{1}a_{2}b_{1}b_{2}}\left(\alpha ,0,\beta ,0,1,1\right),P_{a_{1}a_{2}b_{1}b_{2}}\left(\alpha ,0,0,\beta ,1,2\right),$$

$$P_{a_{1}a_{2}b_{1}b_{2}}\left(0,\alpha ,\beta ,0,2,1\right),P_{a_{1}a_{2}b_{1}b_{2}}\left(0,\alpha ,0,\beta ,2,2\right),$$

where α,β=±1. Thus, model $M_{\mathrm{KH}}$ can be realized with the space of hidden variables consisting on 16 points,

(13) $$\Lambda =\{\left(\alpha ,0,\beta ,0,1,1\right),\left(\alpha ,0,0,\beta ,1,2\right),\left(0,\alpha ,\beta ,0,2,1\right),\left(0,\alpha ,0,\beta ,2,2\right):\alpha_{i},\beta_{j}=\pm 1\}.$$

This space is endowed with probability given by the jpd

### 3.4. Correspondence between Observational and Classical Conditional Probabilities

Now consider the observational probabilities $p_{A_{i},B_{j}}.$ These are probabilities for the fixed pair of experimental settings (i,j). Their counterparts in CP-model $M_{\mathrm{KH}}$ are obtained by conditioning on the fixed values of random variables $r_{A}$ and $r_{B}.$ The rule of correspondence between observational and CP-probabilities is based on the following identification (α,β=±1):

(14) $$p_{A_{i}B_{j}}\left(\alpha ,\beta \right)=P\left(a_{i}=\alpha ,b_{j}=\beta |r_{A}=i,r_{B}=j\right)$$

and

(15) $$p_{A_{i}}\left(\alpha \right)=P\left(a_{i}=\alpha |r_{A}=i\right),\;p_{B_{j}}\left(\beta \right)=P\left(b_{j}=\beta |r_{B}=j\right).$$

Thus,

(16) $$p_{A_{i}B_{j}}\left(\alpha ,\beta \right)=\frac{P(\lambda \in \Lambda :a_{i}\left(\lambda \right)=\alpha ,b_{j}\left(\lambda \right)=\beta ,r_{A}\left(\lambda \right)=i,r_{B}\left(\lambda \right)=j)}{P(\lambda \in \Lambda :r_{A}\left(\lambda \right)=i,r_{B}\left(\lambda \right)=j)}.$$

and

(17) $$p_{A_{i}}\left(\alpha \right)=\frac{P(\lambda \in \Lambda :a_{i}\left(\lambda \right)=\alpha r_{A}\left(\lambda \right)=i)}{P(\lambda \in \Lambda :r_{A}\left(\lambda \right)=i)},$$

(18) $$p_{B_{j}}\left(\beta \right)=\frac{P(\lambda \in \Lambda :b_{j}\left(\lambda \right)=\beta ,r_{B}\left(\lambda \right)=j)}{P(\lambda \in \Lambda :r_{B}\left(\lambda \right)=j)}.$$

This correspondence rule for the “basic observables” is completed by the similar rule for random generators $R_{A}$ and $R_{B}$:

(19) $$p_{R_{A}R_{B}}\left(i,j\right)=P\left(\lambda \in \Lambda :r_{a}\left(\lambda \right)=i,r_{b}\left(\lambda \right)=j\right\}.$$

### 3.5. Violation of the CHSH-Inequality by Conditional Correlations

Conditioning on the selection of experimental settings plays the crucial role. The CP-correlations are based on the conditional probabilities

(20) $$\langle a_{i}b_{j}\rangle \equiv E\left(a_{i}b_{j}|r_{A}=i,r_{B}=j\right)$$

$$=\sum_{\alpha ,\beta =\pm 1}\alpha \beta P\left(a_{i}=\alpha ,b_{j}=\beta |r_{A}=i,r_{B}=j\right).$$

We can form the CHSH linear combination of conditional correlations of RVs:

(21) $$\tilde{B}=\langle a_{1}b_{1}\rangle -\langle a_{1}b_{2}\rangle +\langle a_{2}b_{2}\rangle +\langle a_{2}b_{2}\rangle .$$

It is possible to find such classical probability spaces that

$$|\tilde{B}|>2.$$

Since each conditional probability is also a probability measure and since RVs $a_{i},b_{j}$ take values in [–1, +1], the conditional expectations $E(a_{i}b_{j}|r_{A}=i,r_{B}=j)$ are bounded by 1, so

$$|\tilde{B}|\leq 4.$$

Thus, the common claim on mismatching of the CP-description with quantum mechanics and experimental data was not justified. In principle, one can consider linear combination B composed of correlations $\langle a_{1}b_{1}\rangle$ which are not conditioned on a selection of experimental settings. Such B satisfies the CHSH-inequality. However, such correlations cannot be identified with experimental ones.

### 3.6. Construction of jpd from Observational Probabilities

Correspondence rules (14) and (19) imply

(22) $$P_{a_{1}a_{2}b_{1}b_{2}r_{a}r_{b}}\left(\alpha_{1},\alpha_{2},\beta_{1},\beta_{2},i,j\right)=p_{A_{i}B_{j}}\left(\alpha ,\beta \right)p_{R_{A}R_{B}}\left(i,j\right),\alpha ,\beta =\pm 1.$$

From this equality, we can determine all nonzero components the jpd:

$$p\left(\alpha ,0,\beta ,0,1,1\right)=p_{A_{1}B_{1}}\left(\alpha ,\beta \right)p_{R_{A}R_{B}}\left(1,1\right),p\left(\alpha ,0,0,\beta ,1,2\right)=p_{A_{1}B_{2}}\left(\alpha ,\beta \right)p_{R_{A}R_{B}}\left(1,2\right),$$

$$p\left(0,\alpha ,\beta ,0,2,1\right)=p_{A_{2}B_{1}}\left(\alpha ,\beta \right)p_{R_{A}R_{B}}\left(2,1\right),p\left(0,\alpha ,0,\beta ,2,2\right)=p_{A_{2}B_{2}}\left(\alpha ,\beta \right)p_{R_{A}R_{B}}\left(2,2\right).$$

In model $M_{\mathrm{KH}},$ the jpd is completely determined by observational probabilities. In contrast to $M_{\mathrm{CHSH}},$ there are no counterfactual probabilities.

## 4. (No-)Signaling

### 4.1. No-Signaling in Quantum Physics

In the observational framework for the Bohm–Bell type experiment, the condition of no-signaling is formulated in the probabilistic terms. There is no-signaling, from the *B*-side to the *A*-side, if the *A*-marginals of jpds $p_{A_{i}B_{j}}$

(23) $$M_{ij}\left(\alpha \right)=\sum_{\beta =\pm 1}p_{A_{i}B_{j}}\left(\alpha ,\beta \right),\;i=1,2$$

do not depend on the index j. This notion of no-signaling need not be rigidly coupled to quantum observables. It can be applied to any measurement design in that $A_{i}$ is compatible with both $B_{j},j=1,2,$ but $B_{1}$ and $B_{2}$ are incompatible, i.e., we are not able to perform their joint measurement. No-Signaling from the *A*-side to the *B*-side is defined in the same way.

Quantum mechanics obeys the no-signaling condition. The absence of signaling is one of the mysterious features of this theory. No-Signaling is trivial for CP-models. In the presence of jpd, this is just the consequence of coupling of marginal probabilities with jpd. However, in the absence of jpd (see, e.g., Fine [28,29]), no-signaling has no explanation.

One can say that Fine’s theorem is irrelevant to the considered problem because this theorem presents the CP-characterization of *noncontextual* hidden-variables models. In addition, Bell emphasized (see Section 5 for citation) that quantum mechanics has the contextual structure. However, Mermin rightly remarked [78] that in contextual hidden-variables models no-signaling is as mysterious as in quantum mechanics by itself (italic shrift was added by the authors of the present paper):

“If we do the experiment to measure *A* with B,C,… on an ensemble of systems prepared in the state Ψ and ignore the results of the other observables, we get exactly the same statistics for *A* as we would have obtained had we instead done the quite different experiment to measure *A* with L,M,… on that same ensemble. The obvious way to account for this, particularly when entertaining the possibility of a hidden-variables theory, is to propose that both experiments reveal a set of values for *A* in the individual systems that is the same, regardless of which experiment we choose to extract them from. *Putting it the other way around, a contextual hidden-variables account of this fact would be as mysteriously silent as the quantum theory on the question of why nature should conspire to arrange for the marginal distributions to be the the same for the two different experimental arrangements.”*

By using our CP-model $M_{KH}$, we clarify the meaning of signaling at the level of RVs of this model and then at the level of corresponding observables. We remark that another approach to contextual CP-treating of no-signaling was proposed in a series of papers [32,33,34,35,36,37,38].

### 4.2. No-Signaling as a Condition of Independence of Random Variables

Now we proceed with CP-model $M_{\mathrm{KH}}.$ Let us fix $r_{a}=i.$ For any value $r_{b}=j,$ consider conditional $a_{i}$-marginal

(24) $$m_{ij}\left(\alpha \right)=\sum_{\beta }P\left(a_{i}=\alpha ,b_{j}=\beta |r_{a}=i,r_{b}=j\right),i=1,2.$$

By correspondence rules (14), (19),

(25) $$M_{ij}\left(\alpha \right)=m_{ij}\left(\alpha \right).$$

The marginal $m_{ij}\left(\alpha \right)$ does not depend on the *j*-settings governed by $r_{b}$ under the following assumption:

$I_{a_{i}}$ *The pair of RVs $a_{i},r_{a}$ does not depend on RV $r_{b}.$*

Under this assumption

(26) $$m_{ij}\left(\alpha \right)=P\left(a_{i}=\alpha |r_{a}=i\right).$$

This is the conditional-probability version of no-signaling for $a_{i}.$ To prove equality (26), we first remark

(27) $$m_{ij}\left(\alpha \right)=P\left(a_{i}=\alpha |r_{a}=i,r_{b}=j\right)$$

(since the conditional probability is a probability measure). Hence,

(28) $$m_{ij}\left(\alpha \right)=\frac{P(a_{i}=\alpha ,r_{a}=i,r_{b}=j)}{P(r_{a}=i,r_{b}=j)}=\frac{P\left(a_{i}=\alpha ,r_{a}=i\right)P\left(r_{b}=j\right)}{P\left(r_{a}=i\right)P\left(r_{b}=j\right)}$$

and this proves equality (26).

Now, let us assume that RVs $r_{a}$ and $r_{b}$ are independent. (From the experimental viewpoint, this is the very natural assumption.) Suppose that, for α=±1, the marginal $m_{ij}\left(\alpha \right)$ does not depend on j. Generally, this marginal can be represented in the form:

(29) $$m_{ij}\left(\alpha \right)=P\left(a_{i}=\alpha |r_{a}=i,r_{b}=j\right)=\frac{P(a_{i}=\alpha ,r_{a}=i|r_{b}=j)}{P(r_{a}=i|r_{b}=j)}=\frac{P(a_{i}=\alpha ,r_{a}=i|r_{b}=j)}{P(r_{a}=i)}.$$

The right-hand side does not depend on *j* only if $P\left(a_{i}=\alpha ,r_{a}=i|r_{b}=j\right)=P\left(a_{i}=\alpha ,r_{a}=i\right)$ (see Appendix A). This is the condition of independence of the pair of RVs $a_{i},r_{a}$ from RV $r_{b}$.

In the same way, consider the assumption

$I_{b_{j}}$ *The pair of random variables $b_{j},r_{b}$ does not depend on $r_{a}.$*

Under this assumption,

$$m_{ij}\left(\beta \right)=\sum_{\alpha }P\left(a_{i}=\alpha ,b_{j}=\beta |r_{a}=i,r_{b}=j\right)=P\left(b_{j}=\beta |r_{b}=j\right).$$

This is the conditional version of no-signaling for random variable $b_{j}.$

The CP-presentation of no-signaling in terms of conditional probabilities, see $I_{a},I_{b},$ explains the meaning of signaling. For example, b→a signaling means either interdependence of random generators $r_{a}$ and $r_{b},$ or dependence of *a*-RVs on random generator $r_{b}.$

Under the assumption of independence of RVs $r_{a}$ and $r_{b}$ representing the random generators, b→a signaling has the meaning of dependence of *a*-variables on random generator $r_{b},$ i.e., the latter governs not only *b*-variables, but even the *a*-variables.

### 4.3. Interpretation of No-Signaling: From Random Variables to Observables

By using Equation (29), we can lift the CP-interpretation of no-signaling to the level of observables. Let us consider the case of independent random generators $R_{A}$ and $R_{B}$ represented by independent RVs $r_{a}$ and $r_{b}.$ The absence of B→A signaling for observables, i.e., independence $M_{ij}\left(\alpha \right)$ from index j, is equivalent to the absence of b→a signaling RVs. Hence, *at the observational level B→A, no-signaling has the meaning of independence of A-observables from a selection of experimental settings governed by random generator $R_{B}.$* We stress that $M_{\mathrm{KH}}$ can serve as a CP-model for quantum probabilities, i.e., probabilities described by the quantum formalism with the aid of the Born rule. Thus, the absence of signaling in the quantum description of the Bohm–Bell experiment has very natural CP-explanation: selection of *A*-settings depends only on the random generator $R_{A}$ and selection of *B*-settings depends only on the random generator $R_{B}.$ Thus, *no-signaling can peacefully coexist with contextuality.*

### 4.4. (No-) Signaling in Experiments in Quantum Physics and Psychology

In quantum physics, the problem of the presence of signaling patterns in statistical data collected in the Bohm–Bell type experiments was highlighted in the work [42] (it seems it was the first paper on this problem). Since the quantum formalism predicts the absence of signaling, such signaling patterns were considered as a consequence of the improper experimental performance. After the pioneer paper [42], experimenters started to pay attention to signaling. Tremendous efforts of experimenters to eliminate technicalities which may lead to signaling were culminated in the breakdown experiments of Vienna’s group [43] and NIST’s group [44]. (Unfortunately, the first experiment claiming to be loophole free [45] suffers from strong signaling—see [46]).

As was found by Dzhafarov and the coauthors, see, e.g., [33,36], the psychological experiments of the Bohm–Bell type generated statistical data with statistically non-negligible signaling patters. (These are experiments to test quantum contextuality in the psychological analogs of the Bell–Bohm type experiments [63,66]. Thus, the issue of nonlocality is not involved.) In psychology, we do not have theoretical justification of the absence of signaling. Therefore, it is not clear whether the mental signaling is a consequence of improper experimental design and performance or this is the fundamental feature of experiments with humans.

## 5. Hidden-Variables Models: Noncontextual versus Contextual, Local versus Nonlocal

Hidden variables were introduced in the line with von Neumann’s no-go theorem as representing dispersion free states, see, e.g., Bell [15,16,79] and especially Gudder [80,81,82]. Each value $\lambda_{0}$ of hidden variable λ determines uniquely the values of all observables. Thus, the observables can be mathematically represented as functions of λ. Such hidden-variables models are known as *deterministic*. Mathematically, they are represented by Kolmogorov probability spaces [11], triples of the form (Λ,F,P). Here, Λ is the set of hidden variables, F is a σ-algebra of subsets of Λ, and *P* is a probability measure on F. Observables are represented by RVs, (measurable) functions on Λ. The ranges of values of observables and corresponding RVs should coincide (see Mermin [78] for details). Average of observable *A* which is represented by RV *a* is given by

(30) $$\langle A\rangle \equiv \langle a\rangle =\int_{\Lambda }a\left(\lambda \right)dP\left(\lambda \right).$$

More generally, for any set of compatible (jointly measurable) observables A,B,…,K and any (bounded and measurable) function f, average of the operator-function f(A,B,…,K) is given by

(31) $$\langle f\left(A,B,\ldots ,K\right)\rangle =\int_{\Lambda }f\left(a\left(\lambda \right),b\left(\lambda \right),\ldots ,k\left(\lambda \right)\right)dP\left(\lambda \right).$$

Such hidden-variables models are known as *noncontextual.* Model $M_{BCHSH}$ explored by Bell and Clauser, Horne, Shimony, and Holt (see Section 2.2) is a (deterministic) noncontextual model.

It is well known that Bell argued that “the result of an observation may reasonably depend not only upon the state of the system (including the hidden variables) but also on the complete disposition of the apparatus” [79]. Shimony [83] stressed that this is the first statement about contextuality (although Bell did not use this terminology). Hidden-variables models of such type are known as contextual. In fact, Bell’s statement is closely coupled with Bohr’s emphasis of the role of experimental arrangement. However, Bohr considered quantum mechanics as a complete theory. The state of a system is given by wave function ψ and there is no need in supplementary parameters λ. (We remind that the name “contextualistic” was introduced by Shimony [84] and a shortening to “contextual” was performed by Beltrametti and Cassinelli [85].) Shimony made the Bohr–Bell statement concrete on the role of experimental arrangement as follows [83]:

“John Stewart Bell (1928-90) gave a new lease on life to the program of hidden variables by proposing contextuality. In the physical example just considered, the complete state λ in a contextual hidden variables model would indeed ascribe an antecedent element of physical reality to each squared spin component $s_{n}^{2}$ but in a complex manner: the outcome of the measurement of $s_{n}^{2}$ is a function $s_{n}^{2}\left(\lambda ,C\right)$ of the hidden variable λ and the context C, which is the set of quantities measured along with $s_{n}^{2}.$ ... a minimum constraint on the context *C* is that it consists of quantities that are quantum mechanically compatible, which is represented by self-adjoint operators which commute with each other...”

For a contextual model, average’s representation (31) is modified as follows:

(32) $$\langle f\left(A,B,\ldots ,K\right)\rangle =\int_{\Lambda }f\left(a_{C}\left(\lambda \right),b_{C}\left(\lambda \right),\ldots ,k_{C}\left(\lambda \right)\right)dP_{C}\left(\lambda \right),$$

where context *C* is determined by the set of compatible observables C={A,B,…,K} which are represented by RVs $a_{C},b_{C},\ldots ,k_{C}.$ We continue citation of Shimony [83]:

“Another reasonable constraint on *C* of great conceptual importance was proposed by Bell when the system of interest consists of two or more spatially separated parts, and the physical quantity of interest *A* concerns one of these parts. *C* should not include quantities whose measurements are events with space-like separation from the measurement of A, since there would be a violation of relativistic locality if those measurements affected the outcome of the measurement of A.”

Thus, we have two types of contextuality, local and nonlocal. This local versus nonlocal structure of contextual models with hidden variables is not so much emphasized in modern studies on contextuality. Quantum contextuality is identified with a nonlocal one.

Model $M_{KH}$ is a contextual hidden-variables model. We point out that, by writing paper [31], its author was unaware about original works on contextual hidden-variable models (Gudder [80,81,82], Bell [79], Shimony [83], Mermin [78]). This lack of knowledge led to the statement: “We emphasize that our construction of the classical probability space for the EPR–Bohm–Bell experiment cannot be used to support the hidden variable approach to the quantum phenomena. The classical random parameter involved in our considerations cannot be identified with the hidden variable which is used the Bell-type considerations.” This statement was a consequence of the very restricted picture of hidden-variables models borrowed from the original Bell paper [15] (see also [26]). Our model has three distinguishing features:

1. RVs are context-independent, i.e., the *C*-index can be omitted:
  (33) $$\langle f\left(A,B,\ldots ,K\right)\rangle =\int_{\Lambda }f\left(a\left(\lambda \right),b\left(\lambda \right),\ldots ,k\left(\lambda \right)\right)dP_{C}\left(\lambda \right),$$
2. Contextual probabilities $\left\{P_{C}\right\}$ can be selected as conditional probabilities with respect to a single probability measure $P:P_{C}\left(E\right)=P\left(E|C\right).$ (In particular, contexts have the set-representation and conditional probability is given by Bayes’ formula.)
3. The model is locally contextual.

This is the good place to mention the hidden-variables interpretation of CP-model $M_{DZ}$:

1. RVs are context-dependent, i.e., the *C*-index cannot be omitted.
2. Instead of a family of contextual probabilities $\{P_{C}\},$ one can proceed with a single probability measure P:
  (34) $$\langle f\left(A,B,\ldots ,K\right)\rangle =\int_{\Lambda }f\left(a_{C}\left(\lambda \right),b_{C}\left(\lambda \right),\ldots ,k_{C}\left(\lambda \right)\right)dP\left(\lambda \right).$$
3. The model is nonlocally contextual.

The rest of this section is devoted to analysis of the locality issue. This issue is very complex and it is not basic for the present paper which is devoted to the analysis of the possibility of construction of the CP-representation of quantum probabilities. Therefore, the coming analysis cannot be considered as complete. We come back to it in one of the further publications.

In his seminal paper [16], Bell used the following definition of locality:

Now we make the hypothesis [68], and it seems one at least worth considering, that if the two measure- ments are made at places remote from one another the orientation of one magnet does not influence the result obtained with the other.

This definition matches with Einstein’s viewpoint on locality, see Bell’s citation [16] of Einstein [68]:

But on one supposition we should, in my opinion, absolutely hold fast: the real factual situation of the system S1 is independent of what is done with the system S2, which is spatially separated from the former.

Bell concluded his article [16] with the following statement:

In a theory in which parameters are added to quantum mechanics to determine the results of individual measurements, without changing the statistical predictions, there must be a mechanism whereby the setting of one measuring device can influence the reading of another instrument, however remote. Moreover, the signal involved must propagate instantaneously, so that such a theory could not be Lorentz invariant.

One of the problems with treatment of the locality issue in the Bell-framework is that space-time is absent in Bell’s mathematical formalization (see [86,87] for a discussion). In the following consideration, we shall ignore this problem (consideration of locality without using a mathematical model based on Minkovsky’s space-time, cf. [88].

In the hidden-variables framework, Bell formalized the notion of locality (locality hypothesis) as follows. To make our notation closer to Bell’s notation, denote by a(i,j,λ) and b(j,i,λ) RVs corresponding to measurement of observables $A_{i}$ and $B_{j},$ respectively, under selection of settings $r_{a}=i$ and $r_{b}=j.$ The locality hypothesis is that RV a(i,j,λ) does not depend on the index *j* and RV b(j,i,λ) does not depend on the index *i* (see [67], p. 65, Equations (2) and (3)).

Let us analyze the locality issue for CP-model $M_{\mathrm{KH}}.$ Consider realization of this model based on the space of hidden variables Λ defined in Equation (13). For reader’s convenience, we list these 16 points once again:

(α,0,β,0,1,1),(α,0,0,β,1,2),(0,α,β,0,2,1),(0,α,0,β,2,2),

where $\alpha_{i},\beta_{j}=\pm 1.$ RVs are functions defined on Λ. For α,β=±1, the following equalities hold:

(35) $$a_{1}\left(\alpha ,0,\beta ,0,1,1\right)=a_{1}\left(\alpha ,0,0,\beta ,1,2\right)=\alpha$$

and

(36) $$a_{1}\left(0,\alpha ,\beta ,0,2,1\right)=a_{1}\left(0,\alpha ,0,\beta ,2,2\right)=0.$$

Thus, the values of RV $a_{1}$ representing observable $A_{1}$ do not depend on the values of RV $r_{b}$ ruling selection of experimental settings for $S_{2}$ nor on the values of *b*-RVs representing *B*-observables (“the real factual situation of system $S_{1}$ is independent from what is done with system $S_{2}^{\prime \prime })$.

*The CP-model $M_{\mathrm{KH}}$ is local in the Einstein–Bell sense.*

Model $M_{\mathrm{KH}}$ is locally contextual. It is contextual because the values of RV $a_{i}$ depend on outcomes of RV $r_{a}$ representing observable $R_{a}$ compatible with observable $A_{i}.$

The considered locality condition is the analog of *local causality* or the locality condition as considered by Bell [79]. Typically, these conditions are formulated in the probabilistic terms for stochastic hidden-variables models. The condition of locality is formulated as the *probability factorization condition* [79]. Hidden-variables model $M_{KH}$ is deterministic. However, to proceed closer to the standard framework, we shall also use the probabilistic terminology. We remark that, for a deterministic model, all conditional probabilities are equal either to zero or to one. In our notation, the factorization condition can be written as follows (for α,β=±1):

(37) $$P\left(\alpha ,\beta |r_{a}=i,r_{b}=j,\lambda \right)=P\left(\alpha |r_{a}=i,\lambda \right)P\left(\beta |r_{b}=j,\lambda \right)$$

or

(38) $$\frac{P(a_{i}=\alpha ,b_{j}=\beta ,r_{a}=i,r_{b}=j,\lambda )}{P(r_{a}=i,r_{b}=j,\lambda )}=\frac{P(a_{i}=\alpha ,r_{a}=i,\lambda )}{P(r_{a}=i,\lambda )}\;\frac{P(b_{j}=\beta ,r_{b}=j,\lambda )}{P(r_{b}=j,\lambda )}.$$

Let i=1,j=1 and let λ=(α,0,β,0,1,1). Then,

$$P\left(a_{1}=\alpha ,b_{1}=\beta ,r_{a}=1,r_{b}=1,\lambda \right)=P\left(\lambda \right),\;P\left(a_{i}=\alpha ,r_{a}=1,\lambda \right)=P\left(\lambda \right);P\left(b_{1}=\beta ,r_{b}=1,\lambda \right)=P\left(\lambda \right).\;P\left(r_{a}=1,r_{b}=1,\lambda \right)=P\left(\lambda \right);P\left(r_{a}=1,\lambda \right)=P\left(\lambda \right);P\left(r_{b}=j,\lambda \right)=P\left(\lambda \right).$$

Thus, for this λ,

$$P\left(\alpha ,\beta |r_{a}=1,r_{b}=1,\lambda \right)=1,\;P\left(\alpha |r_{a}=1,\lambda \right)=1,P\left(\beta |r_{b}=1,\lambda \right)=1.$$

In the same way, we consider all cases of matching the indexes of $a_{i}$ and $b_{j}$ with the last digits of λ:

- i=1,j=2 and λ=(α,0,0,β,1,2),
- i=2,j=1 and λ=(0,α,β,0,2,1),
- i=2,j=2, and λ=(0,α,0,β,2,2).

The conditional probabilities on the right-hand and left-hand sides of Equation (37) also equal one.

Now, consider mismatching the indexes of RVs $a_{i}$ and $b_{j}$ with the last digits of λ. For example, let i=1,j=1 and $\lambda =(\alpha ,0,0,\beta^{\prime },1,2).$ Here,

$$P(a_{1}=\alpha ,b_{1}=\beta ,r_{a}=1,r_{b}=1,\lambda )=0,$$

$$P\left(a_{i}=\alpha ,r_{a}=1,\lambda \right)=P\left(\lambda \right);P\left(b_{1}=\beta ,r_{b}=1,\lambda \right)=0,$$

$$P\left(r_{a}=1,r_{b}=1,\lambda \right)=0;P\left(r_{a}=1,\lambda \right)=P\left(\lambda \right);P\left(r_{b}=j,\lambda \right)=0.$$

We extend the definition of conditioning to the case such that both nominator and denominator equal zero. In such a case, we set conditional probability to zero. Thus,

$$P(a_{1}=\alpha ,b_{1}=\beta |r_{a}=1,r_{b}=1,\lambda )=0,$$

$$P\left(a_{1}=\alpha |r_{a}=1,\lambda \right)=1,P\left(b_{1}=\beta |r_{b}=1,\lambda \right)=0.$$

Thus, the factorization condition trivially holds as 0=0.

One may think that this (natural) regularization of conditional probability is the root of violation of Bell’s theorem. This is not the case. Even regularized conditional probability $P(\alpha ,\beta |r_{a}=i,r_{b}=j,\lambda )$ provides the right representation:

(39) $$\sum_{\lambda }P\left(\alpha ,\beta |r_{a}=i,r_{b}=j,\lambda \right)P\left(\lambda \right)=P\left(a_{i}=\alpha ,b_{j}=\beta ,r_{a}=i,r_{b}=j\right).$$

The main issue is the correspondence rule coupling probabilities of model $M_{KH}$ with observational probabilities. The probability on the right-hand side of Equality (39) does not coincide with the observational probability. The latter equals $P(a_{i}=\alpha ,b_{j}=\beta |r_{a}=i,r_{b}=j).$

### 5.1. How Can This Happen?

We pointed to the possibility to violate Bell’s type inequalities in the local contextual framework:

*By rejection of the BCHSH-rule for coupling observational probabilities with CP-probability on the space of hidden variables.*

## 6. Conclusions

The paper contains a brief review on CP-representations of the probabilistic structure of quantum mechanics. The main part of the paper is devoted to one special CP-representation based on the *conditional probability interpretation* of quantum probabilities (see also [31]). The conditional probability approach is presented in a very general setting covering the experimental schemes of the Bohm–Bell type. Such experimental schemes need not be coupled to quantum physics. In particular, they can be realized for experiments with humans. As was found by Dzhafarov and the coathors (see, e.g., [36,65]), the latter experiments are characterized by the presence of statistically significant signaling patterns. In this paper, we analyzed the CP-meaning of signaling in the conditional probabilistic model. We found that signaling can be described simply as dependence on the random variables. Another version of the CP-analysis of the meaning of signaling in the Bohm–Bell type experiments was presented on the basis of model $M_{DZ}$ (see [32,38]).

We highlight the basic impacts of the CP-representation of quantum physics:

1. It demystifies the probabilistic structure of quantum mechanics, namely, the representation of probabilities by complex amplitudes and observables by Hermitian operators:
2. It justifies the use of CP-based mathematical statistics for analysis of data from quantum experiments.
3. It shows the possibility to describe the experimental schemes of the Bohm–Bell type with the aid of local contextual hidden-variables models.

Additionally, our model $M_{KH}$ clarifies the meaning of (no-)signaling as independence– dependence of classical random variables. Its construction also demonstrated that the correlations from the Bohm–Bell type experiments can be described by a local contextual hidden-variables model.

CP-models are not directly coupled to the quantum formalism (including the Hilbert space representation of probabilities). Therefore, they can be used to describe mathematically the experimental schemes of the Bohm–Bell type outside of quantum physics, e.g., in psychology, game theory, and decision-making [32,36,63,65].

Finally, we emphasize once again the foundational impact of Ballentine’s works [48,50] on the conditional probabilistic interpretation of quantum probabilities. These works stimulated development of contextual probability theory [61]. As was found in [31], the quantum contextual probabilities generated in experiments of the Bohm–Bell type can be even represented as classical probabilities (see also [32,33,34,35,36,37,38]).

## Appendix A

Consider two RVs *X* and *Y* Here *X* is an arbitrary discrete RV, $X=x_{1},\ldots ,x_{m},$ and *Y* is a dichotomous RV, Y=1,2. Suppose that, for each x, conditional probability P(X=x|Y=j) does not depend no j. We want to show that this implies that, in fact,

(A1) P(X=x|Y=j)=P(X=x),

i.e., that RVs *X* and *Y* are independent.

Set $A_{x}=\left\{\lambda \in \Lambda :X\left(\lambda \right)=x\right\}$ and $B_{j}=\left\{\lambda \in \Lambda :Y\left(\lambda \right)=j\right\}.$ We have

$$P\left(A_{x}|B_{1}\right)=P\left(A_{x}|B_{2}\right),\;i.e.,\;P\left(A_{x}\cap B_{1}\right)=\frac{P(B_{1})}{P(B_{2})}P\left(A_{x}\cap B_{2}\right),$$

or

$$P\left(A_{x}\cap B_{1}\right)=\frac{P(B_{1})}{P(B_{2})}\left[P\left(A_{x}\right)-P\left(A_{x}\cap B_{1}\right)\right],$$

i.e.,

$$P\left(A_{x}\cap B_{1}\right)\left[1+\frac{P(B_{1})}{P(B_{2})}\right]=\frac{P(B_{1})}{P(B_{2})}P\left(A_{x}\right).$$

Thus, we obtained

$$P\left(A_{x}\cap B_{1}\right)=P\left(B_{1}\right)P\left(A_{x}\right).$$

This also implies that $P\left(A_{x}\cap B_{2}\right)=P\left(B_{2}\right)P\left(A_{x}\right).$ Hence, Equality (A1) holds and RVs *X* amd *Y* are independent.
